# Nearly One in Three Lactating Mothers Is Suffering from Inadequate Dietary Diversity in Amhara Region, Northwest Ethiopia

**DOI:** 10.1155/2020/7429034

**Published:** 2020-09-23

**Authors:** Netsanet Fentahun, Elisabeth Alemu

**Affiliations:** ^1^Department of Nutrition and Dietetics, School of Public Health, Bahir Dar University, Bahir Dar, Ethiopia; ^2^Organization for Rehabilitation and Development of Amhara, Bahir Dar, Ethiopia

## Abstract

**Background:**

In developing countries, dietary diversity is a challenge for rural communities especially pregnancy and lactation. Malnourished mothers are unable to combat disease and feed adequate nutrients to their children, and this will in turn affect the socioeconomic development of the country. To date, there is paucity of evidence regarding predictors of dietary diversity among lactating mothers in developing countries. The main objective of this study was to determine the extent and predictors of dietary diversity among lactating mothers in Lay Gayint District, Amhara Region, Ethiopia.

**Methods:**

A community-based cross-sectional study design was employed on 416 systematically selected lactating mothers from March 1–30, 2018. The data were collected using pretested interviews. Data were entered and analyzed using SPSS version 21 software. Bivariable and multivariable logistic regression models were used to determine the predictors of dietary diversity. Odds ratio with 95% confidence interval and *p* ≤ 0.05 were used to test the association.

**Result:**

A total of 268 (65.7%) lactating mothers had inadequate dietary diversity. Adequate dietary diversity was significantly associated with mobile phone usage (OR: 2.3; 95% CI: 1.15–4.55); practice of home gardening (OR: 4.1; 95% CI: 1.71–9.87); pulses production (OR: 4.8; 95% CI: 2.50–9.32); delivery in health center (OR: 4.7; 95% CI: 1.80–12.25); food secured household (OR: 2.4; 95% CI: 1.25–4.62); three meals per day within the previous seven days (OR: 11.12; 95% CI: 2.74–45.24); and practice of income generating activity (OR: 4.00; 95% CI: 2–8.33).

**Conclusion:**

Meal frequency, home gardening practices, pulses production, delivery at health center, practice of income generating activity, food insecurity, and mobile phone usage had significant association with dietary diversity. Therefore, concerned bodies should design multidimensional livelihood and health service programs to alleviate inadequate dietary diversity.

## 1. Background

Dietary diversity has been a challenge in developing countries, especially in rural communities. In countries where resources are limited, lack of access to adequate and diversified diet has been identified as one of the severe problems among poor populations [1, 2]. Women at reproductive age in low- and middle-income countries are vulnerable to malnutrition. Moreover, maternal nutrient needs increase during pregnancy and lactation. When these needs are not met, women may suffer from malnutrition [3].

Dietary diversity (DD) is one of the proxy indicators for measuring dietary adequacy among individuals [4]. Lack of diversified diets is a severe problem in the developing world. A nondiversified diet can have negative consequences on individuals' health, well-being, and development [5]. Maternal undernutrition is the major challenge of our times, and it has been on the global agenda as central to health, sustainable development, and progress [6]. Maternal undernutrition has an effect on human, social, and economic costs to society such as lost productivity, health, and well-being, decreased learning ability, and reduced fulfillment of human potential [7].

Ethiopia is one of the low-income countries with the highest levels of lactating mothers' malnutrition in sub-Saharan Africa [8, 9]. Similarly, the studies conducted in Aksum and South Gondar [10, 11] showed that DD of lactating mothers were low. However, there is little documentation on predictors of dietary diversity among lactating mothers in Amhara Region, Ethiopia. The aim of the study was to determine the extent and predictors of dietary diversity among lactating mothers in Lay Gayint District, Amhara region, Ethiopia. Identification of the extent and predictors of dietary diversity is significant for designing proper intervention to improve maternal nutrition and easily access information for further research about lactating mothers.

## 2. Methods

### 2.1. Study Setting and Design

A community-based cross-sectional study design was employed in Lay Gayint District, South Gondar Administrative Zone, Amhara National Regional State. The district has 31 kebeles located 237 km from the region capital Bahir Dar. The district has a total population of 206,499 (male: 104,401 and female: 102,098) and 12,984 lactating mothers who have under two children. Out of the total population, 22,825 (11%) are urban dwellers, whereas 183,674 (88.95%) live in rural areas (CSA, 2007) [12], where low health facilities, lack of drinking water, and shortage of dietary diversity are manifested. There are seven governmental health centers and one governmental general hospital in the district. The district is one of the food insecure zones in the region which is characterized by shortage of food, lack of drinking water, and land degradation. Most of the communities are highly dependent on relief or donations. Most of the areas have high land agro ecology [13]. The study was conducted from March 1–30, 2018.

### 2.2. Study Populations, Sampling Size Determination, and Sampling Procedures

The study population was lactating mothers who have children of two and less than two years in the selected smallest administrative units. The sample size is determined using single population proportion formula with the following assumptions: 56.4% of proportion of lactating mothers who have inadequate dietary diversity [10], 95% of the confidence interval, and 5% marginal error. By adding a 10% nonresponse rate, the final sample size was 416. There are 31 rural kebeles in the study district, and based on this, 20% of the total kebeles (6 kebeles) were selected through simple random sampling techniques. Then, the sample size was distributed to each selected lowest administrative levels (kebeles) using proportional allocation to size.

The systematic random sampling technique was used to select lactating mothers. Accordingly, the number of lactating mothers from the sampled kebele was selected in an (*k*) interval which was determined by dividing the total number of lactating mothers to the desired sample size, i.e., *k* = *N*/*n*. Here, the interval is approximately 6 for all sampled kebeles. Finally, the total of every 6th lactating mother in each sampled kebele was included in the study.

### 2.3. Measurements

Dietary diversity score is the sum of nine food groups eaten in the previous 24 hours. Women dietary diversity uses the following nine food groups: starchy staples, dark green leafy vegetables, vitamin A-rich vegetables and fruits, organ meat, flesh meat and fish, eggs, dairy, legumes, and nuts [14]. Dietary diversity is categorized as follows: consumption of foods from ≤3 food groups was considered as low dietary diversity; consumption of foods from 4 to 5 food groups was considered as medium dietary diversity, while consumption of food items from ≥6 food groups in 24 hours prior to the interview will be considered as high dietary diversity [14].

Household food insecurity was measured using a Household Food Insecurity Access Scale (HFIAS) adapted from household food insecurity scales that were previously validated for use in developing countries. It is the measure of the degree of food insecurity in the household in the past 4 weeks (30 days). It consists of two types of related questions. The first question type is called an occurrence question. There are nine occurrence questions that ask whether a specific condition associated with the experience of food insecurity ever occurred during the previous 4 weeks (30 days). Each severity question is followed by a frequency of occurrence question, which asks how often a reported condition occurred during the previous 4 weeks. There are three response options representing a range of frequencies (1 = rarely; 2 = sometimes; 3 = often). Finally, individuals were classified as food secure if the individuals responded “no” to all of the items and insecure if the individuals responded “yes” to at least one of the nine items [15].

### 2.4. Data Quality

The questionnaires had been pretested in Lay Gayint District at Sali rural kebele to check on the length, content, question wording, and language. This allows modifications on the questionnaires by correcting mistakes and inclusion of foods that may have been missed out or elimination of foods that may not be applicable in the community. Ambiguous questions were corrected to ensure clarity of the required information and then to enhance reliability. Furthermore, in order to control the quality of the data; correction and modification were done based on the gap identified during interview. Grade 12 and diploma holders were recruited as data collectors and supervised by clinical nurses. Training was given on the aim of the research, content of the questionnaire, and how to conduct interview and supervise to increase their performance in field activities. The collected data were checked every day by supervisors, and the authors checked the completeness and consistency.

### 2.5. Data Analysis

Data were entered and analyzed using by SPSS version 21. Proportions and summary statistics were performed and bivariable and multivariable logistic regressions were used to determine the predictors of adequate dietary diversity. The variables that were found with *p* < 0.2 at bivariable logistic regression were entered to multivariable analysis and those variables with *p* value <0.05 were considered statistically significant. The Hosmer–Lemeshow goodness-of-fit test was used to assess the fitness of the model.

### 2.6. Ethical Considerations

Ethical clearance was obtained from institutional review board of Bahir Dar University. Permission letter was obtained from the concerned bodies of the district and kebele administrations through a formal letter. The nature of the study was fully explained to the study participants to obtain their oral consent, and confidentiality of the information was ensured through not writing and recording the name of respondents, interviewing them privately. Study participants were also informed that they had a full right not to participate in the study and could withdraw any time.

## 3. Results

### 3.1. Sociodemographic Characteristics of Lactating Women

A total of 408 lactating women were included in the study. Most of the lactating women (72.8%) were in the age group of 15–30 years old. Above half of the lactating women (57%) were unable to read and write. Most of the lactating women were married (92%). Greater than half of the lactating women (65%) had four to six family sizes (Table 1).

### 3.2. Socioeconomic Characteristics of Lactating Women

Most of the lactating women (85%) had own production for their food source as well as possessed farm lands (88%). Lactating women had low home gardening practice (19.4%) while pulse production was found to be better compared to practice of home gardening (44.9%). Three-fourth of the lactating women reported that they possessed big animals while half of them were found to possess small animals. Furthermore, a quarter of the lactating women had additional income generating activities. Regarding access to information and communication, half of the lactating women had a mobile phone.

### 3.3. Work Burden of Lactating Women

From the total lactating women, 66% faced shortages of time to prepare food. And among them, 20%, 51%, and 49% lactating women gave care for members of the family, did extra outdoor activities (such as shopping, fetching water, and collecting firewood), and did farming activities in the field, respectively. Concerning the working hour, 65.7% of lactating women reported that they had worked 9 to 12 hours per day. Because of being busy, 38% of the participants reported that they spend time without food. Concerning fetching water, 14.5% of lactating mothers reported that they have spent more than 60 minutes to fetch water (Table 2).

### 3.4. Health Service Utilization

Three hundred fifty-two (86%), 383 (94%), and 318 (78%) of the participants reported that they used antenatal care, postnatal care, and delivered at health center, respectively. 190 (51%) of the participants reported that there is illness within two weeks. From HFIAS, 58% of the respondents reported they worried about food; 63% of them faced lack of preferred food; 64% of them had limited access to a variety of foods; and 29% of them were forced to eat unpreferred food due to lack of resources.

### 3.5. Food Insecurity and Meal Frequency

Out of the total lactating mothers of Lay Gayint District, 163 (40%), 1724 (30%), 62 (15.19%), 29 (7%), and 16 (4%) of the respondents reported that they were eating small portions, they were skipping meals, their households ran out of food, they went to sleep hungry, and they remained 24 hours without food, respectively. Regarding the food security status of lactating mothers, 286 (70%) of lactating mothers are food insecure. Out of the total lactating mothers, 261 (64%) of the participants reported that they had consumed three meals per day within the past 7 days. The mean meal frequency of lactating mothers was three (+SD 0.64).

### 3.6. Dietary Diversity of Lactating Mothers

Nearly all women (99.5%) consumed starchy staples, and 90.7% of them consumed pulses and 94.6% of them did not have any green leaf vegetation in previous 24 hours. More than two-third of the participants (71.1%) reported that they did not have fruits and vegetables within 24 hours, and 62% of the participants indicated that they had fat and oil within 24 hours (Table 3).

### 3.7. Predictors of Lactating Mothers' Dietary Diversity

Lactating women who possessed a mobile phone were 2.3 times more likely (OR: 2.3; 95% CI: 1.15–4.54) to have a diversified diet compared to those who did not have the mobile phone. Lactating women who practiced home gardening were four times more likely (OR: 4.1; 95% CI: 1.70–9.86) to have a diversified diet than those who did not practice. Lactating women who produced pulses were 4.8 times more likely (OR: 4.8; 95% CI: 2.50–9.32) to diversify their diet compared to those who did not. Lactating women who delivered in health center were 4.7 times more likely (OR: 4.7; 95% CI: 1.79–12.25)) to have diversified diet compared to their counterparts. Lactating women who had food secured households were 2.4 times more likely (OR: 2.4; 95% CI: 1.24–4.62) to have diversified diet compared to those who have food insecure household. Lactating women who had above three meals per day within the previous seven days were 11 times more likely (OR: 11.1; 95% CI: 2.73–45.24) to have diversified diet compared to those who had below three meals per day within the previous seven days. Lactating women who had a practice of income generating activities were four times more likely (OR: 4.0; 95% CI: 2–8.33)) to have diversified diet compared to those who did not practice income generating activities (Table 4).

## 4. Discussion

This study tried to determine the predictors of dietary diversity among lactating mothers in Lay Gayint District, Amhara National Regional State, Ethiopia. The prevalence of inadequate dietary diversity was 65.7% in this study. This is a very alarming proportion that could show most of the lactating mothers and their children are at risk of malnutrition. A study conducted in Aksum town showed that about 56% lactating mothers had low dietary diversity [10]. This is somehow a better figure as compared to the result of the present study. However, the current study was a community-based cross-sectional study at rural area while the pervious study was in urban area where better life style is expected.

Large proportions of the lactating women in the study area seem to have good access to carbohydrates and fats and oils. However, only little proportion could access protein (meat, fish, egg, and dairy products) and vitamin sources within 24 hours. This can be strong evidence that the study population is exposed to malnutrition in terms of protein. This finding is almost consistent with the finding of other similar studies conducted in various developing countries [16, 17] as well as in different localities in Ethiopia [10, 18]. In fact, cereals such as maize, wheat, teff, and barley are the dominantly grown crops in the study areas [19]. In addition, palm oil is occasionally provided for the community.

The most important predictors of dietary diversity were meal frequency, pulses production, institutional delivery, home gardening, and income generating activity. Thus, immediate interventions may be needed towards alleviating low dietary diversity by encouraging the society to regularly practice the above factors. This study showed that mothers lactating more than three times per day are 11.12 times more likely to diversify their diet. Similarly, a study conducted in Aksum reported a strong association between meal frequency and dietary diversity [10].

Lactating mothers who produced pulses were 4.8 times more likely to diversify their diet. Pulses are main crops to fulfill protein energy requirement in the world. Of course, those who did not produce pulses always eat by buying the pulses by compromising other food items. In a study conducted in Alamata area, pulses production was not associated with dietary diversity [20]. This might be due to the difference in the food security status of the study population, which was almost food secured. In addition, difference in other socioeconomic variables between the two study populations can bring variation.

Lactating mothers who delivered in health center were 4.7 times more likely to diversify their diet. This result agrees with EDHS [21] data. This is directly related with the education provided on antenatal care (ANC) regarding nutritional counseling.

Lactating mothers who practiced home gardening were 4 times more likely to have a diversified diet. This is consistent with studies conducted in Aksum [10] and in South Gondar Zone [11]. Indeed, there is a clear association between having a home garden and a more varied diet [22, 23]. Households having gardens could use surplus vegetables and fruits from their gardens as a means to diversifying their daily food. In addition, they have better income from selling of vegetables and fruits, which can be used to purchase other food items.

Lactating mothers who had a practice of income generating activity were 4.45 times more likely to diversify their diet compared to those who did not practice IGA. Studies conducted in rural Burkina Faso [24] and in some other low-income countries [4] have shown similar result with the current study. Many other studies in low- and middle-income countries have already indicated strong association between higher socioeconomic status and high diet diversity score. Socioeconomic well-being of the participants had also strong association with dietary diversity. A study conducted in South Gondar Zone on household dietary diversity [11] showed that off-farm activity was a predictor of household dietary diversity. However, income generating activity means additional income source besides their main households' livelihood. Especially, in food secured households, income generating activity supports household expenditure during a shortage of annual product. This, in turn, is used to fulfill minimum dietary diversity and compensate the gap of dietary diversity, which might be having a significant association with DD. Lactating mothers who had food secured household were 2.4 times more likely to diversify their diet compared to those who have food insecure household. In this study, the proportion of food insecurity among the lactating mothers was very high (70%). In fact, the study area is one of the 64 districts in Amhara Region which is reported to lose their productivity due to land degradation and subjected to food insecurity [24]. A study conducted in Kenya [17] and other low-income countries [4] showed that food security had association with dietary diversity.

However, the study conducted in Aksum town [10] and a comparative study of lactating mothers in Raya Alamata [20] revealed that there was no association between food security and dietary diversity. Such differences between the current study result and that in Aksum and Alamata are due to the prevalence of food insecurity status in Aksum (29%) and Alamata (2–4%), participants' residential address, study design, and socioeconomic status of the participants in the study areas.

Lactating mothers who had a mobile phone were 2.2 times more likely to have a diversified diet compared to their counterparts. Same results were reported by previous study conducted in South Gondar [11] and in Alamata [20]. The possible explanation might be that participants who had a mobile phone have better economic well-being and develop purchasing and exchanging power to diversify their diet.

## 5. Conclusions

Meal frequency, home gardening practices, pulses production, delivery at health center, practice of income generating activity, food insecurity, and mobile phone usage had significant association with dietary diversity. Therefore, concerned bodies should design multidimensional livelihood and health service programs to alleviate inadequate dietary diversity.

## Figures and Tables

**Table 1:** Sociodemographic characteristics of lactating mothers in Lay Gayint District (*n* = 408), 2018.

Variables	Frequency	%
Age of lactating mothers (years)		
15–30	297	73
31–40	98	24
>40	13	3

Age of children (month)		
0–6	99	24
9–12	143	35
>12	166	41

Lactating mothers' educational status		
Unable to read and write	232	57
Able to read and write (informal education)	37	9
Grade 1–6	53	14
Grade 7–10	75	18
>Grade 10	11	2

Marital status of lactating women		
Married	374	92
Divorce	18	4
Single	11	3
Widowed	5	1

Husband education		
Unable to read and write	146	36
Able to read and write (informal education)	122	35
Grade 1–6	37	9
Grade 7–10	68	17
>Grade 10	12	3

Family size		
1–3	88	21
4–6	264	65
>6	56	14

**Table 2:** Work burden of lactating mothers (*n* = 408) in Lay Gayint District, South Gondar Zone, 2018.

Lactating women activities	Frequency	Percent
Give care for members of the family		
Yes	81	20
No	327	80

Time shortage to prepare food		
Yes	176	43
No	232	57

Extra outdoor activities		
Yes	271	66
No	137	34

Field/farm work		
Yes	208	49
No	200	51

Presence of assistant to cook food		
Yes	70	17.4
No	338	82.6

Presence of supporter to fetch water		
Yes	169	41.4
No	239	58.6

Working hour per day		
≤8 hours	109	26.7
9 to 12	268	65.7
>12	31	7.6

Time spent without eating food due to being busy		
Yes	155	38
No	253	62

Time spent to fetch water		
<5 and 5 minute	41	10
6 to 30 minute	271	66.4
31–60 minute	37	9.1
>60 minute	59	14.5

Time spent for queuing to fetch water		
0–15 minute	107	26.5
16–45 minute	46	12
45–120 minute	103	25.5
>120 minute	142	36

**Table 3:** Dietary diversity frequency of lactating mothers (*n* = 408) in Lay Gayint District, South Gondar Zone, Ethiopia, March 2018.

Food group consumed within the previous 24 h		Frequency	Percent
Carbohydrates and starch	Yes	406	99.5
	No	2	05

Green leafy vegetable	No	386	94.6
	Yes	22	5.4

Sources of vitamin A	No	387	94.9
	Yes	21	5.1

Fruits and vegetables	No	290	71.1
	Yes	118	28.9

Fat and oil	No	156	38
	Yes	252	62

Meat and fish	No	378	92.6
	Yes	30	7.4

Egg	No	379	92.9
	Yes	29	7.1

Pulse	No	38	9.3
	Yes	370	90.7

Dairy products	No	383	94
	Yes	25	6

Dietary diversity (DD)	Low	268	65.7
	Medium	123	30.1
	High	17	4.2

**Table 4:** Bivariable and multivariable associations of factors with dietary diversity among lactating mothers in Lay Gayint District, South Gondar Zone, Ethiopia, March 2018.

Variables	Dietary diversity		COR (95% C.I.)	AOR (95% C.I.)
	Adequate	Inadequate		
Mobile phone				
Yes	103	104	4.6 (2.945, 7.272)	2.29 (1.152–4.546)
No	165	36	1	1

Home gardening				
Yes	18	61	10.7 (5.98, 19.22)	4.1 (1.707–9.866)
No	250	79		1

Pulses production				
Yes	78	105	7.3 (4.592, 11.629)	4.8 (2.508–9.325)
No	105	35		

Income generating activities				
Yes	50	85	6.35 (4.23, 10.019)	4 (2–8.331)
No	218	57		

Delivered in health center				
Yes	191	127	3.94 (2, 7.388)	4.7 (1.799–12.250)
No	77	13		

Household food insecurity status				
Food secured	50	72	4.6 (2.938, 7.254)	2.4 (1.245–4.620)
Food insecure	218	68	1	1

Meal frequency				
Below 3 meals	66	5	1	1
3 meals	190	71	4.9 (1.909, 12.743)	2.5 (0.746–8.458)
>3 meals	15	61	70.4 (23.470, 211.170)	11.12 (2.735–45.243)

Adequate dietary diversity includes both medium and high dietary diversity categories; inadequate dietary diversity includes low dietary diversity.

## Data Availability

The datasets supporting the conclusions of this article are included within the article.

## Abbreviations

ANC: Antenatal care
BOFED: Bureau of Finance and Economic Development
DDS: Dietary diversity score
DD: Dietary diversity
FANTA-2: Food and Nutrition Technical Assistance II Project
FAO: Food and Agriculture Organization of the United Nations
FFQ: Food frequency questionnaire
FSNP: Food security and nutrition policy
GOE: Government of Ethiopia
IDDS: Individual dietary diversity score
IFPRI: International Food Policy Research Institute
EDHS: Ethiopia Demographic and Health Survey
ENBS: Ethiopia National Bureau of Statistics
MDG: Millennium development goals
PNC: Postnatal care
UNICEF: United Nations International Children's Emergency Fund
HFIAS: Household Food Insecurity Access Scale
WRA: Women of reproductive age
WHO: World Health Organization
WFP: World Food Program.
